# A New Theory for the Evolution of Genomic Imprinting

**DOI:** 10.1371/journal.pbio.0040421

**Published:** 2006-11-14

**Authors:** Liza Gross

When Watson and Crick solved the structure of DNA in 1953, the mechanism of heredity was immediately apparent in the pairing of the nucleotide bases. And the history of life, it appeared, could be inferred by decoding the messages written in their sequence. But it has become increasingly clear that heritable changes in gene expression can occur without alterations in DNA sequence. These “epigenetic” mechanisms can alter gene activity by chemically modifying the DNA or the proteins that envelop it.

One epigenetic mechanism, called genomic imprinting, has proven especially puzzling, because it appears to undermine the benefits that multicellular organisms gain from inheriting two copies, or alleles, of nearly every gene. (If one allele is damaged, the activity of the other can often compensate.) In genomic imprinting, only the gene inherited from one parent is expressed; the other is silenced by a chemical “stamp,” thereby forfeiting the advantage of having two alleles. Errors in imprinting have been linked to cancer and some genetic diseases. Why would selection favor a “mono-allelic” expression pattern for genes that exposes the organism to genetic injury?

In a new study, Jason Wolf and Reinmar Hager address these issues with a new theory for the evolutionary origins of genomic imprinting. Providing an alternative to the dominant model, the authors show that the expression of maternally derived genes allows for the coadaptation of complementary traits between mother and offspring and enhances offspring development and fitness.

The dominant model explains asymmetric parental gene expression in terms of conflict. In one scenario, the conflict arises over maternal investment, such that paternally expressed growth factor genes, for example, would require more maternal investment during development while maternally expressed growth inhibitors would require less, with different implications for offspring fitness. Alternately, genomic imprinting might mitigate intralocus sexual conflict—which occurs when gender-specific selection favors different alleles in males and females—by allowing high-fitness alleles to pass from mothers to daughters and from fathers to sons.

**Figure:**
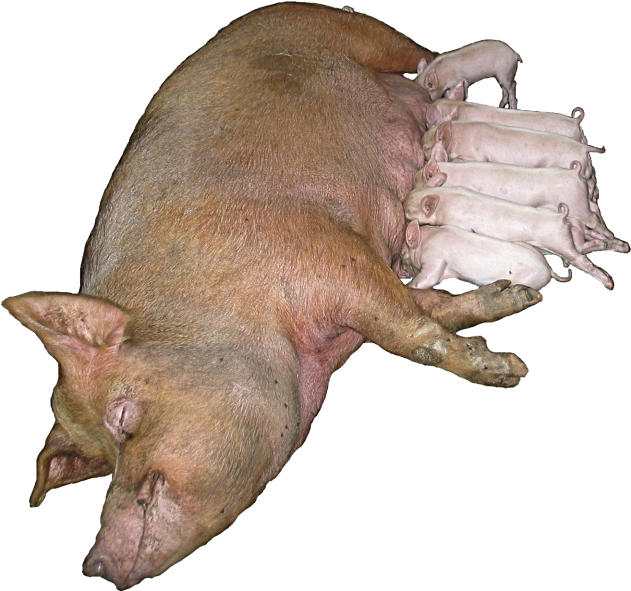
Natural selection favors the sole expression of maternal gene copies, because it allows for the genetic coadaptation of maternal offspring traits and leads to higher offspring fitness.

But these conflict theories don’t account for evidence of genetic coadaptation between mother and offspring—based on correlations between offspring begging behavior in birds and maternal response, for example. The authors’ model does account for such evidence, however, by demonstrating that when selection favors such coadaptation between maternal and offspring traits, evolution may lead to maternal expression at genomic loci underlying these traits. Coadaptation could arise through two different selection modes: pleiotropy and linkage disequilibrium. In pleiotropy, one genomic locus with two alleles affects both the maternal and offspring trait—a condition the authors explored through a single-locus model. In linkage disequilibrium, trait-related alleles are linked in the genome; this case was explored in a two-locus model in which two separate loci (each with two alleles) affect the maternal and offspring trait. Both models assume that selection favors coadaptation by linking offspring fitness to the combined genomic expression of mother and offspring.

To determine whether either model favors the evolution of genomic imprinting, the authors mathematically analyzed the relationship between the level of imprinting and the average fitness of individuals. Imprinting will be favored, they found, when genetic variation exists for coadapted traits. Since genetic variation for maternal and offspring traits “appears ubiquitous” in natural populations, it’s likely that this variation influences the evolution of imprinting, the authors conclude. Genomic imprinting increases population mean fitness, they explain, by increasing the adaptive melding of maternal and offspring traits.

How does the theory play out in practice? A recent study in mice showed that every gene that is exclusively imprinted in the placenta was maternally expressed—suggesting the genes’ critical role in placental development—and supporting the model’s prediction that genes involved in “intimate maternal-offspring interaction” are more likely to show maternally expressed imprinting. This prediction can be experimentally tested in organisms for which such intimate interactions have significant fitness implications, such as plant-eating insects. In this case, offspring survival depends on where the mother deposits the eggs, and one would expect to see genetic coadaptation for traits affecting oviposition site and offspring performance.

Though their study focused on maternal-offspring interactions, the authors expect coadaptation to occur between father and offspring when the father is the primary caregiver, which describes many fish and arthropod species. Their theory also provides researchers with a roadmap for testing alternative hypotheses for the origins and targets of genomic imprinting—which are likely to vary with the taxa under study.

